# Association between neutrophil-to-lymphocyte ratio and early renal function decline in patients with immunoglobulin a nephropathy

**DOI:** 10.1080/07853890.2025.2559133

**Published:** 2025-10-23

**Authors:** Cun Shen, Mengchao Liu, Zhen Cai, Mengdi Wang, Lei Tian, Long Tang, Fangqiang Cui, Wenjing Zhao

**Affiliations:** Department of Nephropathy, Beijing Hospital of Traditional Chinese Medicine, Capital Medical University, Beijing, China

**Keywords:** IgA nephropathy, neutrophil-to-lymphocyte ratio, estimated glomerular filtration rate, early renal function decline

## Abstract

**Background:**

IgA nephropathy (IgAN) is a chronic immune-mediated progressive kidney disease. The Neutrophil-To-Lymphocyte Ratio (NLR) has emerged as a novel marker for systemic immune inflammation. This study aimed to explore the relationship between NLR and early renal function decline in patients with IgAN.

**Methods:**

A real-world cross-sectional observational study was conducted on a cohort of 1073 patients diagnosed with IgAN. Patients were stratified into high and low NLR groups based on a predefined cut-off value. We examined the association between NLR levels and reduced renal function across these groups, defined as an estimated glomerular filtration rate (eGFR) of 60 mL/min·1.73 m^2^ or less, using receiver operating characteristic (ROC) curve analysis and Logistic regression models.

**Results:**

Among the 1,073 patients evaluated, those with an eGFR ≤ 60 mL/min·1.73 m^2^ had significantly higher NLR levels compared to those with eGFR > 60 mL/min·1.73 m^2^ (*Z* = 9.598, *p* < 0.001). The area under the AUROC for NLR was 0.671 (*p* = 0.000, 95% *CI*: 0.638–0.703). The high NLR group showed significantly elevated levels of mean arterial pressure, 24-hour urinary protein and uric acid, along with lower hemoglobin, serum albumin, and eGFR (all *p* < 0.001). Multivariate logistic regression analysis revealed that high NLR was independently associated with reduced eGFR (≤ 60 mL/min·1.73 m^2^) (*p* = 0.009, *OR* = 2.110, 95% *CI*: 1.208–3.683). A nomogram was developed to identify IgAN patients with reduced eGFR (≤60 mL/min·1.73 m^2^).

**Conclusions:**

IgAN patients with higher NLR levels exhibited more severe clinical manifestations. A high NLR may be associated with an increased risk of eGFR ≤ 60 mL/min·1.73 m^2^ in patients with IgAN.

## Introduction

IgA nephropathy (IgAN) is the most common form of primary glomerulonephritis globally [1], exhibiting significant heterogeneity with 30-50% of patients progressing to end-stage renal disease (ESRD) within 5 to 10 years [2,3]. Identifying individuals at the highest risk for progression to ESRD remains a significant challenge. The 2021 Kidney Disease Improving Global Outcomes (KDIGO) guidelines have emphasized risk stratification as a crucial research area in IgAN and recommended an international prediction tool for IgAN. This tool is designed to help predict the risk of a 50% reduction in glomerular filtration rate (GFR) or the development of ESRD following a renal biopsy [4]. A GFR of 60 mL/min·1.73 m^2^ represents a critical threshold, indicating the onset of renal dysfunction and compensation failure. At this stage, patients may experience complications and face significantly increased side effects from hormonal or immunosuppressive treatments. Therefore, it is imperative to identify IgAN patients at high risk for early renal function decline.

IgAN is characterized by the accumulation of IgA-dominant deposits within the glomeruli, with the inflammatory responses and immune reactions playing pivotal roles in its onset and progression [5]. The specific immunoinflammatory markers involved in IgAN have yet to be fully defined. Current risk prediction tools primarily focus on clinical renal indicators and histopathological findings such as Oxford MEST-C scores. However, these factors alone often fail to capture the dynamic role of immune-inflammatory processes in disease progression, limiting prognostic accuracy, especially for identifying high-risk patients who could benefit from early intervention. Given the link between mucosal immunity, infections, and IgAN progression, markers like the neutrophil-to-lymphocyte ratio (NLR) may provide additional prognostic value by reflecting disease activity and renal injury.

NLR serves as a biomarker reflecting the inflammatory response and is a marker of poor prognosis in several disorders, including chronic kidney disease (CKD), malignancies, and myocardial infarction [6,7,8,9]. An increase in neutrophil count or a decrease in lymphocyte count results in an elevated NLR, which typically indicates an ongoing inflammatory reaction. Activated neutrophils infiltrate the kidneys in IgAN, releasing reactive oxygen species, myeloperoxidase, and pro-inflammatory cytokines (e.g. IL-6, TNF-α), exacerbating glomerular and tubulointerstitial damage. Neutrophil extracellular traps (NETs) is upregulated in IgAN and plays a key role in its pathogenesis by promoting inflammatory cytokine release. Inhibition of NETosis improves both clinical and pathologicaloutcomes [10]. Galactose-deficient IgA1 (Gd-IgA1) also activates B cells to produce autoantibodies, perpetuating immune complex formation [11]. CD4+ T-cell subsets (Th1/Th17) drive inflammation by secreting IFN-γ and IL-17, while Treg depletion impairs immune tolerance [12]. Thus, NLR reflects the imbalance between pro-inflammatory neutrophils and dysregulated lymphocytes, offering a composite measure of immune-mediated renal injury in IgAN. Elevated NLR may predict worse outcomes by capturing this synergistic pathology. Elevated NLR also heightens the risk of ESRD through several mechanisms, including its influence on treatment efficacy. Specifically, a higher NLR is associated with suboptimal therapeutic responses, potentially contributing to disease progression and an unfavorable prognosis [13]. Although direct studies examining the relationship between NLR and prognosis in IgAN are limited, some retrospective analyses and cohort studies have indicated that NLR may be a risk factor for ESRD in patients with IgAN, particularly those with stage 3 to 4 chronic kidney disease or 24-hour urinary protein of > 1 g/day [14]. However, the association between NLR and reduced eGFR (≤ 60 mL/min·1.73 m^2^) in IgAN remains uncertain. To address this gap, we conducted a retrospective cross-sectional study involving 1,073 patients with IgAN to provide further evidence of the association between NLR and early renal function decline. Furthermore, we explored whether incorporating NLR into an IgAN clinical risk model could improve the assessment of early renal function decline at baseline.

## Patients and methods

### Study design and participants

This retrospective study was approved by the Ethics Committee of Beijing Hospital of Traditional Chinese Medicine, Capital Medical University (2023BL02-022-02) and was conducted under the ethical principles outlined in the Declaration of Helsinki. Due to the retrospective cross-sectional design of the research, the ethics committee approved a waiver of informed consent. This exemption was justified as the study involved exclusively anonymized and de-identified data.

**Figure 1.**
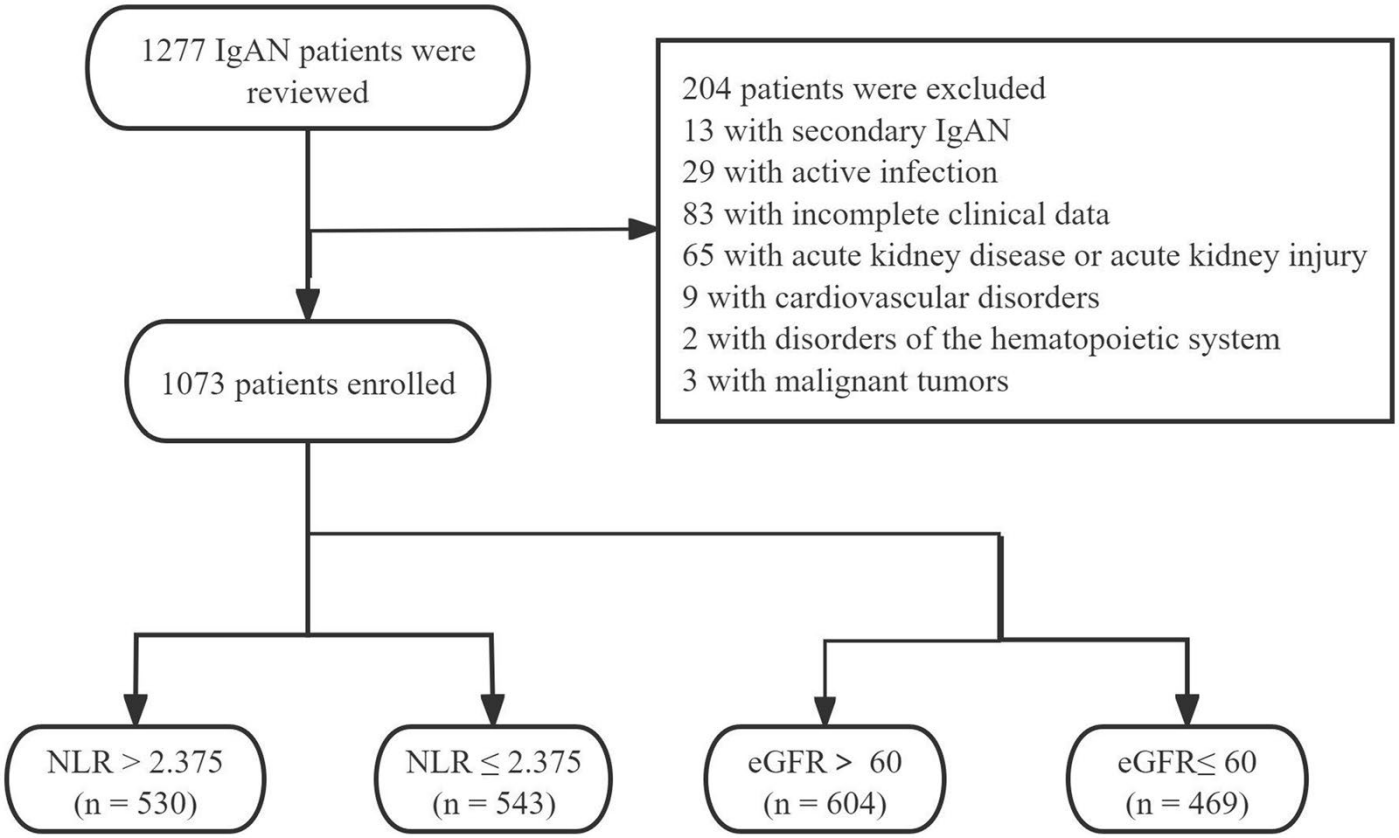
Flowchart showing the number of IgAN patients included in the analyses.

We included 1277 patients diagnosed with primary IgAN *via* biopsy, who were monitored from January 1, 2010, to December 31, 2023, at the Beijing Hospital of Traditional Chinese Medicine, affiliated with Capital Medical University in Beijing, China. 204 individuals were excluded for the following reasons: (1) incomplete clinical data; (2) presence of secondary IgAN induced by conditions such as systemic lupus erythematosus or anaphylactoid purpura; (3) acute kidney disease or acute kidney injury; and (4) severe comorbid conditions including infectious diseases, cardiovascular disorders, disorders of the hematopoietic system, or malignant tumors (Figure 1).

### Covariate collection and assessments

Data analyzed in this study were sourced from the scientific research sharing platform (Yidu Cloud Research Collaboration Platform) of the Beijing Hospital of Traditional Chinese Medicine affiliated to Capital Medical University. All participants completed standardized questionnaires to collect demographic and medical information. Clinical data were collected either at the time of renal biopsy or during the initial patient encounter and included variables such as sex, age, mean arterial pressure (MAP), serum albumin (sALB), serum creatinine (SCr), estimated glomerular filtration rate (eGFR), uric acid (UA), total cholesterol (TC), triglycerides (TG), high-density lipoprotein cholesterol (HDL-C), low-density lipoprotein cholesterol (LDL-C), 24-hour urinary protein, neutrophil count (NE), lymphocyte count (LY), platelet counts (PLT), hemoglobin (HGB), and C-reactive protein (CRP). All tests were performed twice before reporting and were conducted by trained staff at the laboratory of the Beijing Hospital of Traditional Chinese Medicine, affiliated with Capital Medical University.

The eGFR was calculated using the Chronic Kidney Disease Epidemiology Collaboration (CKD-EPI) equation during the initial patient encounter [15]. Participants were stratified into two groups based on whether their eGFR exceeded 60 mL/min·1.73 m^2^. Additionally, the Neutrophil to Lymphocyte Ratio (NLR) and the Platelet to Lymphocyte Ratio (PLR) were computed. The Systemic Immune-Inflammation Index (SII) was determined based on the formula: Neutrophil Count × Platelet Count/Lymphocyte Count.

### Statistical analysis

All statistical analyses were conducted using IBM SPSS software, version 29.0. Data following a normal distribution were presented as mean ± standard error (mean ± SE), and differences between groups were assessed using the independent-samples t-test. Data with a skewed distribution were described by the median and interquartile ranges [M (Q1, Q3)], and the Mann-Whitney U rank sum test was employed for comparisons. Categorical data were reported as frequency and composition ratio [N (%)], with the chi-square test (χ^2^) used to test associations. Using an eGFR of 60 mL/min·1.73 m^2^ as the classificating threshold, receiver-operating characteristic (ROC) analysis was utilized to evaluate the association between the NLR, PLR, and SII and early renal function decline (eGFR ≤ 60 mL/min·1.73 m^2^) in patients with IgAN. The optimal cut-off values were determined based on the maximum Youden index (Youden Index = Sensitivity + Specificity − 1). Furthermore, multivariate logistic regression analysis was performed to identify potential risk factors for eGFR ≤ 60 mL/min·1.73 m^2^ in patients with IgAN. A nomogram was constructed using the “RMS” package within the R programming environment (version 3.5.2). Statistical significance was established at a two-sided *P*-value of less than 0.05.

## Results

### Baseline characteristics

The baseline characteristics of the 1,073 IgAN patients at the time of renal biopsy or initial visit are described in Table 1. The median age of the cohort was 53 years, with an interquartile range (IQR) of 42 to 63 years. The distribution by sex was nearly even, with 522 (48.6%) being male and 551 (51.4%) female. Patients were categorized into two groups: one group with an eGFR > 60 mL/min·1.73 m^2^ (*n* = 604, 56.3%) and another with an eGFR ≤ 60 mL/min·1.73 m^2^ (*n* = 469, 43.7%). Comparative analysis of clinical data between the two eGFR groups revealed significant differences in all measured clinical indices, except for sex, MAP, NE, CRP, TG, TC, and LDL-C.

**Table 1.** Clinical characteristics of patients with IgAN according to eGFR.

Characteristics	Total (*n* = 1073)	eGFR> 60 (*n* = 604)	eGFR≤ 60 (*n* = 469)	*t/Z/χ*^2^ Value	*P* Value
Male (%)	522 (48.6)	282 (46.7)	240 (51.2)	2.125	0.145
Age (year)	53 (42, 63)	48 (38,60)	55 (47, 66)	10.222	**<0.001**
Mean arterial pressure (mmHg)	93.3 (88.0, 100.0)	90.3 (84.3, 96.7)	96.7 (91.0, 103.3)	7.211	**<0.001**
24 h urinary protein (mg/24h)	1179.3 (514.0, 2271.0)	727.5 (286.0, 1528.8)	1398.0 (710.0, 2784.6)	11.200	**<0.001**
NE (10^9^/L)	4.37 (3.50, 5.65)	4.35 (3.51, 5.63)	4.38 (3.49, 5.70)	0.938	0.348
LY (10^9^/L)	1.53 (1.17, 1.96)	1.85 (1.51, 2.12)	1.35 (1.02, 1.76)	−10.424	**<0.001**
PLT (10^9^/L)	216 (180, 266)	238 (208, 286)	197 (164, 251)	−8.221	**<0.001**
HGB (g/L)	128.1 ± 21.7	137.2 ± 16.8	116.5 ± 21.8	16.998	**<0.001**
NLR	2.82 (2.05, 3.95)	2.39 (1.81, 3.16)	3.35 (2.40, 4.37)	9.598	**<0.001**
PLR	129.8 (103.5, 165.5)	124.8 (99.5, 158.9)	138.0 (111.4, 175.3)	13.836	**<0.001**
SII	627.7 (462.0, 875.1)	606.1 (433.2, 855.1)	646.9 (468.0, 926.7)	3.821	**<0.001**
CRP (mg/L)	1.9 (1.2, 3.8)	2.00 (1.20, 3.50)	1.90 (1.18, 4.30)	1.134	0.257
sALB (g/L)	36.8 (32.7, 40.1)	41.0 (38.0, 43.3)	37.8 (34.1, 41.3)	−9.010	**<0.001**
UA (μmol/L)	395.3 ± 103.7	363.6 ± 88.0	436.2 ± 108.1	−11.818	**<0.001**
eGFR (mL/min·1.73 m^2^)	46.2 (11.9, 83.1)	93.6 (72.0, 107.1)	14.3 (7.9, 40.1)	−28.130	**<0.001**
TG (mmol/L)	1.58 (1.16, 2.44)	1.78 (1.29, 2.50)	1.51 (1.11, 2.33)	−0.684	0.494
TC (mmol/L)	4.74 (4.1, 5.8)	4.72 (4.14, 5.84)	4.77 (4.01, 5.86)	0.286	0.775
HDL-C (mmol/L)	1.19 (1.01, 1.42)	1.27 (1.04, 1.43)	1.16 (0.99, 1.40)	−3.898	**<0.001**
LDL-C (mmol/L)	2.74 (2.23, 3.52)	2.73 (2.25, 3.72)	2.74 (2.22, 3.36)	0.230	0.818

**Notes:** Continuous variables are expressed as mean ± standard deviation or median (interquartile spacing). Categorical variables are expressed as count (percentage). Comparisons were made between groups using the *t* test or *χ^2^* test or Wilcoxon rank sum test (if applicable). The presence of bold values indicates that the difference between the two groups for this variable was statistically significant (*p* < 0.05).

**Abbreviations:** MAP, mean arterial pressure; NE, neutrophil count; LY, lymphocyte; PLT, platelet; HGB, hemoglobin; CRP, C-reactive protein; PLT, platelete; NLR, ratio of neutrophil to lymphocytes; PLR, ratio of platelet to lymphocyte; SII, Systemic immune-infammation index; eGFR, estimated glomerular filtration rate; sALB, serum albumin; UA, uric acid; TC, total cholesterol; TG, triglycerides; HDL-C, high density lipoprotein cholesterol; LDL-C, low density lipoprotein cholesterol.

### Diagnostic utility of immunoinflammatory markers for identifying eGFR ≤ 60 mL/min·1.73 m^2^ in IgAN

**Figure 2.**
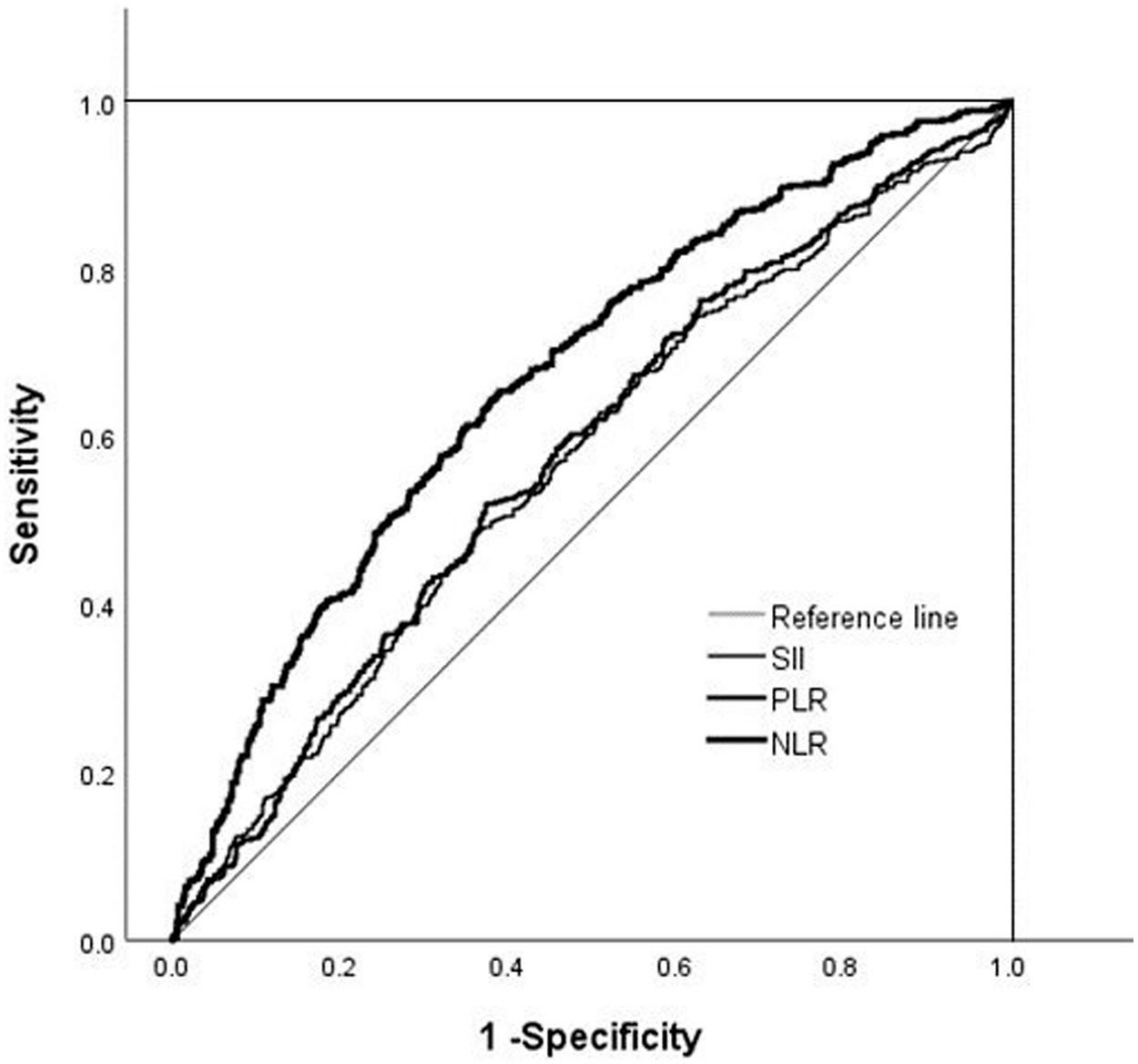
ROC curves showing the sensitivity and specificity of multiple peripheral blood inflammatory indicators for eGFR ≤ 60 mL/min·1.73 m^2^ in IgAN. Among neutrophil-to-lymphocyte ratio (NLR), platelet-to-lymphocyte ratio (PLR) and systemic immune-infammation index (SII), NLR had the largest area under the curve (AUC = 0.671; PLR: 0.578, SII: 0.568).

To assess the diagnostic performance of NLR, PLR, and SII for eGFR≤ 60 mL/min·1.73 m^2^ in IgAN patients, ROC curve analysis was conducted, and the area under the ROC curve (AUC) was calculated for each marker. The AUCs were as follows: NLR at 0.671 (95% CI, 0.638–0.703, *p* = 0.000), PLR at 0.578 (95% CI, 0.544–0.612, *p* = 0.000), and SII at 0.568 (95% CI, 0.533–0.602, *p* = 0.000), as shown in Table 2. Among these biomarkers, NLR demonstrated the highest diagnostic accuracy for identifying eGFR ≤ 60 mL/min·1.73 m^2^ in IgAN patients, as indicated by the highest AUC (0.671) (Figure 2). Consequently, subsequent analyses predominantly focused on clarifying the relationship between NLR and IgAN.

**Table 2.** The area under the ROC curve.

Variables	Cut-Off	AUC	std. Errors	95% CI	Sensitivity(%)	Specificity(%)	*p* Value
NLR	2.375	0.671	0.016	0.638–0.703	65.5	60.9	0.000
PLR	136.393	0.578	0.018	0.544–0.612	51.800	62.6	0.000
SII	612.640	0.568	0.018	0.533–0.602	48.6	63.9	0.000

### Association between NLR levels and clinical variables

According to the cut-off value derived from the Youden index, IgAN patients were stratified into a high NLR group (NLR > 2.375) and a low NLR group (NLR ≤ 2.375). This categorization was employed to investigate the association between NLR and the principal clinical features of IgAN (Table 3). The analysis revealed that patients in the high NLR group displayed more severe clinical manifestations compared to those in the low NLR group. These included a higher MAP (median 94.0, IQR 90.0–101.7 vs. median 91.4, IQR 85.2–96.7, *p* < 0.001), lower HGB (median 126, IQR 110–142 vs. median 131, IQR 119–143, *p* < 0.001), higher 24-hour urinary protein (median 1308.5, IQR 546.5–2744.0 vs. median 847.0, IQR 399.5–1555.0, *p* < 0.001), lower sALB (median 36.6, IQR 31.9–39.8 vs. median 38.8, IQR 34.8–41.9, *p* < 0.001), higher UA (median 405.2, IQR 332.3–481.2 vs. median 369.8, IQR 320.4–434.9, *p* < 0.001), and lower eGFR (median 31.9, IQR 9.4–69.8 vs. median 73.5, IQR 44.1–97.8, *p* < 0.001).

**Table 3.** The comparison between the high and low NLR group in IgAN patients.

Characteristics	IgAN (*n* = 1073)	Low NLR group (*n* = 530)	High NLR group (*n* = 543)	*Z* value	*P* value
NLR levels	2.39 (1.84, 3.31)	1.83 (1.56, 2.07)	3.30 (2.71, 4.21)	28.353	<0.001
MAP (mmHg)	93.3 (86.8, 99.9)	91.4 (85.2, 96.7)	94.0 (90.0, 101.7)	4.077	<0.001
HGB (g/L)	129.0 (115.0, 143.0)	131 (119, 143)	126 (110, 142)	−4.408	<0.001
24 h urinary protein (mg)	1147.4 (482.8, 2185.3)	847.0 (399.5, 1555.0)	1308.5 (546.5, 2744.0)	7.378	<0.001
sALB (g/L)	37.5 (33.4, 40.9)	38.8 (34.8, 41.9)	36.6 (31.9, 39.8)	−6.715	<0.001
UA (μmol/L)	386.0 (325.1, 454.4)	369.8 (320.4, 434.9)	405.2 (332.3, 481.2)	5.052	<0.001
eGFR (mL/min·1.73 m^2^)	50.6 (13.3, 86.6)	73.5 (44.1, 97.8)	31.9 (9.4, 69.8)	−8.743	<0.001

**Notes:** Continuous variables are expressed as median (interquartile spacing).

### Logistic regression analysis of independent risk factors associated with eGFR ≤ 60 mL/min·1.73 m^2^ in IgAN

Initially, univariate logistic regression analysis was conducted on indicators that displayed statistically significant differences between the eGFR > 60 mL/min·1.73 m^2^ group and the eGFR ≤ 60 mL/min·1.73 m^2^ group. This analysis identified 13 potential risk factors associated with reduced eGFR in IgAN. Subsequently, multivariate logistic regression analysis revealed that age, MAP, 24-hour urinary protein, HGB, UA, and NLR are independently associated with eGFR ≤ 60 mL/min·1.73 m^2^ in this cross-sectional cohort of IgAN patients (*p* < 0.05, Table 4).

**Table 4.** Independent risk factors for eGFR ≤ 60 mL/min·1.73 m^2^ in IgAN.

	*β* value	SE value	*Wald χ^2^* value	*OR* value	*95%*CI	*P* value
Age, year	0.039	0.010	15.880	1.040	1.020-1.060	**<0.001**
MAP	0.040	0.015	6.700	1.041	1.010-1.073	**0.010**
24 h urinary protein	0.000	0.000	6.151	1.000	1.000-1.000	**0.013**
HGB	−0.063	0.009	54.145	0.939	0.923-0.955	**<0.001**
UA	0.006	0.001	15.649	1.006	1.003-1.009	**<0.001**
HDL-C	−0.438	0.358	1.498	0.645	0.320-1.301	0.221
NLR(> 2.375; ≤ 2.375)	0.746	0.283	6.896	2.110	1.208–3.683	**0.009**

**Notes:** In multivariate Logistic regression analysis, the presence of bold values indicates that the difference between the two groups for this variable was statistically significant (*p* < 0.05).

**Abbreviations:** β, regression coefficient; SE, standard error; OR, odds ratio; 95% CI, 95% confidence interval.

### Nomogram prediction model

**Figure 3.**
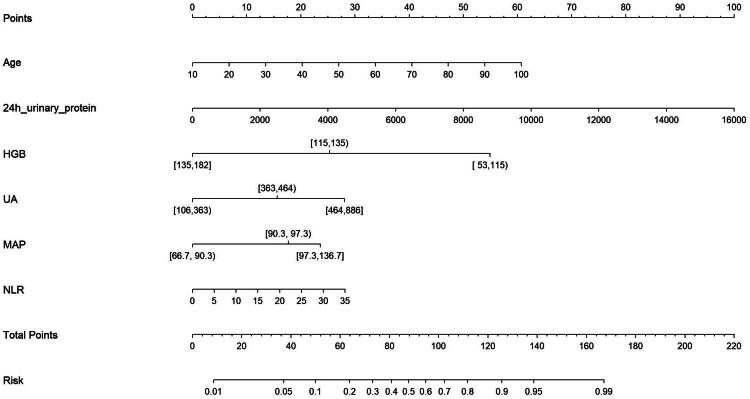
Nomogram prediction models for IgA nephropathy with eGFR ≤ 60 mL/min·1.73 m^2^.

To enhance clinical decision-making, we developed a nomogram prediction model for early renal function decline (eGFR ≤ 60 mL/min·1.73 m^2^) in patients with IgAN, as illustrated in Figure 3. The model’s accuracy was substantiated by a C-index of 0.889 (*p* < 0.001), indicating a significant improvement in model performance.

A hypothetical scenario involving a 40-year-old male patient is considered to illustrate how the model functions: age (20 points), MAP of 95 mmHg (17.5 points), 24-hour urinary protein of 2000 mg (12.5 points), hemoglobin (HGB) at 113 g/L (55 points), UA at 520 µmol/L (27.5 points), and NLR at 3 (2.5 points). With a cumulative score of 135, this yields an estimated probability of renal function decline (eGFR ≤ 60 mL/min·1.73 m^2^) in IgAN of approximately 93.33%.

## Discussion

While the pathogenesis of IgAN remains incompletely understood, the autoimmune inflammatory response is recognized as a crucial pathophysiological mechanism. The “four-hit hypothesis” describes the inflammatory cascade initiated in IgAN: it starts with the formation of Gd-IgA1 in the glomerular mesangium (Hit 1), which precipitates the production of autoantibodies (Hit 2). Subsequent formation and deposition of immune complexes in the mesangium (Hit 3) trigger mesangial cell activation, production of extracellular matrix, activation of the complement system, and release of cytokines and chemokines. Finally, local inflammation and fibrosis occur (Hit 4) [11,16], leading to glomerular injury and disruption of the glomerular filtration barrier. Consequently, the hallmark clinical manifestations of IgAN emerge, namely, hematuria, proteinuria, and progressive kidney dysfunction. Thus, immune mechanisms and chronic inflammation play significant roles in the initiation and progression of IgAN [5]. Despite this understanding, there remains a notable deficit in immunoinflammatory markers for adequately assessing IgAN.

Traditionally, serum creatinine, proteinuria, and the Oxford classification have been recognized as significant risk factors for high-risk IgAN [17]. In this study, we evaluated several immunoinflammatory markers in 1,073 IgAN patients and discovered that LY and PLT counts, along with NLR, PLR, and SII, exhibited significant differences between patients with eGFR > 60 mL/min·1.73 m^2^ and those with eGFR ≤ 60 mL/min·1.73 m^2^. Notably, while LY and PLT counts remained within normal ranges, NLR emerged as the most effective diagnostic tool and an independent risk factor for eGFR ≤ 60 mL/min·1.73 m^2^ in IgAN patients. NLR, which measures the ratio of neutrophils to lymphocytes, reflects the balance between inflammatory agents and regulatory cells. Neutrophils are primarily involved in acute inflammation, while lymphocytes can release inflammatory cytokines and mediate a variety of immune reactions. Therefore, an elevated NLR may indicate heightened immune-related inflammation. NLR has been identified as a novel immunoinflammatory marker in various pathologies, including atherosclerosis [18], hepatocellular carcinoma [19], type 2 diabetes mellitus, and diabetic nephropathy [20]. While previous research has linked high NLR with an increased risk of ESRD [14], its association with decreased eGFR, particularly eGFR ≤ 60 mL/min·1.73 m^2^, remains underexplored, which highlights the importance of early IgAN assessment.

The current study highlights the superior predictive capability of the NLR over the PLR and the SII for IgAN, demonstrating the highest AUC of 0.671. Patients were stratified into two groups based on the NLR threshold of 2.375, which yielded a sensitivity of 65.5% and a specificity of 60.9%. These findings align with previous research where NLR similarly exhibited the highest AUC of 0.695 [12]. Moreover, a higher NLR among IgAN patients was linked to more severe clinical manifestations. Notably, in the high NLR group, MAP, 24-hour urinary protein, UA, and SCr levels were elevated, whereas HGB, sALB, and eGFR were decreased. Previous studies reinforce the role of NLR in clinical severity and prognosis. For instance, adult patients with IgA vasculitis and high NLR levels exhibited more severe clinical symptoms and acute histological features, along with a poorer renal prognosis [12]. Furthermore, NLR has been identified as negatively correlated with eGFR and positively correlated with urinary albumin excretion, particularly in the early stages of renal dysfunction and albuminuria among diabetic patients [21]. Elevated NLR levels have been significantly associated with diabetic nephropathy, suggesting its utility both as a predictive and prognostic risk marker [22]. For example, Yoshtomi et al. demonstrated that a higher NLR was linked to worse renal outcomes, confirming its prognostic value [23]. Similarly, Yuan et al. proposed the use of NLR in risk assessment for patients with stage 4 CKD [24].

In clinical settings, it has been observed that some IgAN patients exhibit a progressive decline in renal function despite well-managed proteinuria and blood pressure. Additionally, while informative, kidney biopsies are invasive and do not offer real-time disease monitoring capabilities. Extensive research has focused on developing risk models for predicting progression to ESRD in IgAN, but models for early renal function decline, specifically to eGFR ≤ 60 mL/min·1.73 m^2,^ have received less attention. Aligning with the KDIGO guidelines for CKD, our study shifts the focus from ESRD to the early stages of renal function decline. This cross-sectional study employed multivariate logistic regression to ascertain that NLR serves as an independent immunoinflammatory marker associated with early renal function decline in IgAN patients. For instance, in a cohort of 271 patients with hypertension and stage 1–3 CKD, those in stage 3 exhibited higher NLR levels compared to stages 1–2 [25]. Further, a comprehensive study involving over 1,000 IgAN patients identified NLR as an independent risk factor for disease progression. The optimal cut-off value for NLR was established at 2.40, with patients above this threshold displaying more severe disease manifestations and poorer prognoses [26]. Moreover, multivariate linear regression analysis revealed that NLR [β = 1.230, 95% CI (0.081, 2.379), *p* = 0.036] might independently influence renal tubular atrophy and interstitial fibrosis in IgAN, with an optimal NLR threshold of 3.25 [27]. Additionally, a longitudinal study by Shang-Feng Tsai et al. which followed 272 IgAN patients over a decade, found that an NLR > 2.75 was an independent predictor of all-cause mortality [28]. These findings demonstrate the utility of NLR as a readily accessible marker for disease progression and a promising immune-related inflammatory biomarker in IgAN patients. However, given its moderate predictive accuracy (AUROC: 0.671), NLR should be interpreted alongside clinical parameters and established IgAN biomarkers (e.g. Gd-IgA1, complement levels) to optimize risk stratification.

Additionally, this study developed a nomogram risk assessment model based on the findings from a multifactorial regression analysis. The evaluation of this model demonstrated statistically significant associations with the presence of reduced renal function (eGFR ≤ 60 mL/min·1.73 m^2^) in patients with IgAN. Accordingly, a nomogram was constructed incorporating key factors including age, MAP, 24-hour urinary protein, HGB, UA, and NLR. This nomogram allows for the individualized assessment of the risk of eGFR ≤ 60 mL/min·1.73 m^2^ through an easy-to-use visual scoring system in IgAN patients. Our findings suggest that while NLR has moderate predictive value alone, its integration into comprehensive assessment models significantly improves individualized risk stratification for early renal function decline in IgAN patients. This supports the potential clinical utility of NLR as part of a multi-parameter evaluation system rather than as an isolated biomarker. The robust performance of our nomogram (C-index 0.889) represents a meaningful step toward personalized medicine in IgAN management, addressing the critical need for early identification of high-risk patients while utilizing readily available clinical data. This approach successfully balances scientific innovation with practical clinical application.

Nonetheless, our study has several limitations that warrant mention. First, it was conducted in a single center, focusing solely on Chinese patients with biopsy-proven IgAN. Consequently, the findings may not be generalizable to other racial or ethnic groups. Second, the study’s cross-sectional design limits our ability to establish causal relationships, and further prospective studies with larger sample sizes are necessary to validate the predictive value of NLR for identifying eGFR ≤ 60 mL/min·1.73 m^2^. Third, additional detailed analyses of inflammatory confounding factors, renal pathologic lesions and medication information are required in future research.

## Conclusion

This study demonstrated that IgAN patients with eGFR ≤ 60 mL/min·1.73 m^2^ often have elevated NLR levels, which might signify more severe clinical manifestations. An increased NLR is associated with early renal function deterioration in IgAN. Crucially, a high NLR could serve as a significant immunoinflammatory factor of eGFR ≤ 60 mL/min·1.73 m^2^ in patients with IgAN.

## Data Availability

The datasets used and/or analyzed during the current study are available from the corresponding authors on reasonable request.
